# Halogenation of Peptides and Proteins Using Engineered Tryptophan Halogenase Enzymes

**DOI:** 10.3390/biom12121841

**Published:** 2022-12-08

**Authors:** Barindra Sana, Ding Ke, Eunice Hui Yen Li, Timothy Ho, Jayasree Seayad, Hung A. Duong, Farid J. Ghadessy

**Affiliations:** 1 Disease Intervention Technology Laboratory, Institute of Molecular and Cellular Biology, Agency for Science, Technology and Research (A*STAR), 8A Biomedical Grove, #06-04/05 Neuros/Immunos, Singapore 138648, Singapore; 2 Institute of Sustainability for Chemicals, Energy and Environment, A*STAR, 8 Biomedical Grove, Neuros, #07-01, Singapore 138665, Singapore

**Keywords:** halogenation, protein, peptide, PyrH, enzyme engineering

## Abstract

Halogenation of bioactive peptides via incorporation of non-natural amino acid derivatives during chemical synthesis is a common strategy to enhance functionality. Bacterial tyrptophan halogenases efficiently catalyze regiospecific halogenation of the free amino acid tryptophan, both in vitro and in vivo. Expansion of their substrate scope to peptides and proteins would facilitate highly-regulated post-synthesis/expression halogenation. Here, we demonstrate novel in vitro halogenation (chlorination and bromination) of peptides by select halogenase enzymes and identify the C-terminal (G/S)GW motif as a preferred substrate. In a first proof-of-principle experiment, we also demonstrate chemo-catalyzed derivatization of an enzymatically chlorinated peptide, albeit with low efficiency. We further rationally derive PyrH halogenase mutants showing improved halogenation of the (G/S)GW motif, both as a free peptide and when genetically fused to model proteins with efficiencies up to 90%.

## 1. Introduction

Numerous peptide-based therapeutics have recently been developed for diverse indications including diabetes, infectious disease, and oncology [1,2,3,4]. Peptide drugs typically comprise non-canonical amino acids with chemical modifications that dramatically alter their physicochemical properties and efficacy [5,6,7]. Notably, halogenation can impart numerous desirable properties [8,9,10], including increased cell membrane and blood-brain–barrier permeability [11,12,13], improved cytotoxicity [14], enhanced target affinity [15], selectivity gains, and reduced side effects [16,17]. Similarly, halogenation can impart dramatic changes in protein functionality [11,18,19]. Halogenated peptides are typically made by incorporation of chemically synthesized amino acid analogues. The viability of post-synthesis in vitro peptide halogenation has not been widely explored, although in vivo post-translational peptide modification is common [8,20]. These modifications are carried out by specific enzymes that could potentially work in vitro. However, studies suggest that these enzymes have co-evolved orthogonally with specific peptide substrates, limiting their application as generic peptide halogenation tools [10,21]. Tryptophan halogenases (TH) catalyse halogenation of the free amino acid tryptophan, both in vitro and in vivo [10,22,23,24]. TH family members are classified based on their regiospecificity towards tryptophan (5, 6 or 7 positions of the indole ring). Extensively studied representatives are *Streptomyces rugosporus* PyrH [25,26] (5 position), *Streptomyces toxytricini* SttH [27] and *Streptomyces violaceusniger* ThHal [28] (6 position), *Pseudomonas fluorescens* PrnA [29] and *Lechevalieria aerocolonigenes* RebH [30] (7 position). These have been engineered extensively to alter regioselectivity and improve activity towards non-natural chemical substrates [23,31,32,33]. Enzymatic halogenation of tryptophan has been coupled to chemocatalytic Suzuki–Miyara cross-coupling, yielding useful derivatives [34,35]. Extrapolation of this approach to peptides and proteins could open up a new method for peptide/protein modification. Here, we show for the first time that TH enzymes can additionally halogenate C-terminal tryptophan residues of synthetic peptides in a context-dependent manner. We further improve this novel activity by structure-guided engineering and utilize these TH variants to site-specifically halogenate model proteins.

## 2. Materials and Methods

The peptides were custom synthesized by Mimotopes (Melbourne, Australia) and Synpeptide Co., Ltd. (Shanghai, China) and purified to >90% purity. Genes and primers were supplied by Integrated DNA Technologies, Singapore. Competent cells and restriction enzymes were obtained from New England Biolabs (Singapore). The reagents and cofactors for halogenation reactions were purchased from Sigma Aldrich (Singapore). Solvents were obtained from Sigma Aldrich and were used without further purification.

**Cloning and enzyme engineering:** The halogenase genes (PyrH, SttH, ThHal, PrnA and RebH) and the flavin reductase gene (RebF) were codon-optimized for recombinant expression in *E. coli*, and cloned using NdeI and XhoI restriction sites of pET22b vector. Uniprot accession numbers for the encoded proteins are, respectively, A4D0H5, E9P162, A0A1L1QK36, P95480, Q8KHZ8 and Q8K176. The constructs were transformed into chemically competent *E. coli* DH5α cells, and the sequences of the constructs were confirmed by DNA sequencing.

The PyrH Q160N mutant was made by PCR-based site directed mutagenesis using 5′- AGC ACC TTG GCA GAG AAT CGT GCA CAA TTC CCT TAC -3′ and 5′- GTA AGG GAA TTG TGC ACG ATT CTC TGC CAA GGT GCT -3′ primers. The PCR product was DpnI digested and transformed into chemically competent *E. coli* DH5α cells. The dASQV mutant was developed by inverse PCR of the wild type PyrH construct (using 5′- GAC GAA TCC CTG GGT CGT AGC ACC TTG -3′ and 5′- GAA TAA TGA TCC ATC AAG CAT ACG TGG TGC GCG TT -3′ primers) followed by DpnI digestion, gel purification and intramolecular ligation of the PCR product. The intramolecular ligation product was transformed into chemically competent *E. coli* DH5α cells. The correct mutations were confirmed by DNA sequencing.

Codon optimized genes of eGFP, Stoffel fragment of Taq DNA polymerase and SpyCatcher with N-terminal His-Tag were cloned using NdeI and BamHI restriction sites of pET22b vector. Genetically encoded LEVLFQGPDYKDDDDK-GGW/-SGW residues were inserted at C-terminus by inverse PCR using the primers in Table S2 (amino acid sequence shown in Table S1). The constructs were transformed into chemically competent *E. coli* DH5α cells, and their sequences were confirmed by DNA sequencing.

**Protein expression and purification:** The correct plasmid constructs (PyrH, SttH, ThHal, PrnA, RebH and RebF genes in pET22b vector) were transformed into *E. coli* BL21(DE3) competent cells, following manufacturer’s protocol (NEB) and grown overnight at 37 °C in LB-agar plates containing suitable antibiotic. Overnight cultures were prepared by inoculating single colony in antibiotic-supplemented LB broth and grown at 37 °C with constant shaking. For overexpression, the overnight cultures were diluted 100-fold in the same media, grown at 37 °C until the OD600 reached 0.5, and induced with 0.5 mM IPTG. The cells were harvested after culturing overnight at 25 °C and stored at −80 °C.

The cells were re-suspended in HisTrap buffer A (50 mM Tris-Cl + 500 mM NaCl + 20 mM imidazole, pH 7.4) and lysed by sonication. Cell debris and insoluble materials were removed by centrifugation at 20,000× *g* for 30 min. The proteins were purified using His GraviTrap Ni-NTA column (GE healthcare) following manufacturer’s protocol, and the protein was eluted in buffer B (50 mM Tris-Cl + 500 mM NaCl + 500 mM imidazole, pH 7.4). The purified proteins were concentrated and buffer exchanged into 50 mM phosphate buffer pH 7.2 using 10 kDa MWCO Amicon Ultra centrifugal filters. Concentration of the purified proteins was determined by OD_280_ using Nanodrop and their purity was analyzed by SDS-PAGE (Figure S36).

**Halogenation reactions:** For peptide halogenation 2.5 mM substrate was treated with 10 µM enzyme and 50 mM NaCl/NaBr in the presence of the cofactors 10 µM FAD, 2 mM NADH and 30 µM flavin reductase enzyme RebF, 20 mM glucose and 5 unit glucose dehydrogenase enzyme. The reaction was carried out in 10 mM phosphate buffer (pH = 7.2) overnight at room temperature with constant mixing. The enzymes were inactivated by heating at 95 °C for 10 min and removed by centrifugation at 13,500 rpm for 10 min. The supernatant was analyzed by LC-MS, and the halogenated and non-halogenated products were quantified from the HPLC peak area% of starting material and product (Figures S26–S33).

For kinetic study, various concentrations (0.5–20 mM) of GGW peptide were halogenated by PyrH-WT and PyrH-Q160N enzymes following the above protocol. The reactions were carried out for 60 min, and samples were collected at 0, 5, 15, 30 and 60 min; and the reactions were stopped immediately by inactivating the enzymes at 95 °C for 10 min and the precipitates were removed by centrifugation at 13,500 rpm for 10 min. The supernatant was analyzed by LC-MS. The amounts of halogenated products were quantified from the HPLC peaks using a standard curve made with various concentration of chlorinated GGW peptides, and used for calculating the reaction rate. The substrate concentrations and the initial reaction rates were used for making Michaelis-Menten plot (Figure S34), which was used to calculate the kinetics parameters.

For protein chlorination, 86 µM protein was treated with 10 µM enzyme and 50 mM NaCl in the presence of the cofactors 10 µM FAD, 2 mM NADH and 30 µM flavin reductase enzyme RebF, 20 mM glucose and 5 unit glucose dehydrogenase (GDH) enzyme. The reaction was done in 10 mM phosphate buffer (pH = 7.2), for 3 h at room temperature with constant mixing. Precipitates, if any, were removed by centrifugation at 13,500 rpm for 10 min. To cleave the halogenated C-terminus, the supernatant was mixed with PreScission protease (10 unit/mL final concentration) and incubated overnight at 4 °C with constant mixing. The reaction mixtures were heated to 95 °C for 10 min, precipitates were removed by centrifugation at 13,500 rpm for 10 min. The supernatant was analyzed by LC-MS, and the halogenated and non-halogenated cleaved C-terminus was quantified from the respective peak area% (Figures S8–S25). To rule out any possible halogenation of cleaved C-terminus peptide during protease treatment at 4 °C, a control experiment was done using the thermostable Stoffel fragment with C-terminal SGW tags (Table S1) where the reaction mixture was heat treated (20 min at 65 °C) to inactivate the PyrH, RebF and GDH enzymes after 3 hours’ halogenation prior the PreScission protease treatment.

**Detection of halogenation position:** The halogenation position was detected in the chlorinated products of two peptides G_5_W and G_3_SGW. The peptides were chlorinated in preparative scale by scaling up the above reaction and the chlorinated products were purified by semi-preparative HPLC. The halogenation site was identified by ^1^H NMR.

**Suzuki coupling reaction:** Peptide GGGSGW-Cl (2.5 mg, 0.0045 mmol), 1,4-benzenediboronic acid bis(pinacol) ester (3 mg, 0.0090 mmol), potassium phosphate tribasic monohydrate (5.2 mg, 0.0226 mmol), Na_2_PdCl_4_/sSPhos, L/Pd = 2:1 stock solution (10 mM, 90 µL) and 30 µL of 1,4-dioxane were added to a microwave vial with a magnetic stir bar. The vial was capped and irradiated with continuous microwave for 3 h at 150 W. The temperature was ramped from RT to 100 °C and kept constant for the entire duration. The reaction mixture was allowed to cool, diluted with water and analyzed via LC-MS.

**Analytical methods:** Chemicals and anhydrous solvents were obtained from Sigma Aldrich and were used without further purification. Spectroscopic grade solvents were purchased from Sigma Aldrich. NMR spectra were recorded on Bruker Avance III 400 MHz spectrometer in MeOD-d4. Data are reported in the following order: chemical shifts are given (δ); multiplicities are indicated as s (singlet), d (doublet), t (triplet), q (quartet) and m (multiplet). High-resolution mass spectra (HRMS) were recorded on an Agilent ESI-TOF mass spectrometer at 3500 V emitter voltage. Exact *m*/*z* values are reported in Daltons.

Analytical UPLC-MS was accomplished by using an Agilent 1290 Infinity II LC system coupled with Agilent 6120 Single Quadrapole MS. 20 μL of crude mixture was injected onto an Agilent EclipsePlus C18 analytical column (1.8 μ packing, 2.1 mm × 50 mm). Gradient starting conditions of 10% (*v*/*v*) MeCN/H_2_O (plus 0.1 % (*v*/*v*) HCOOH) were held for 1 min before development to 95% (*v*/*v*) MeCN/H_2_O over 3 min prior to re-equilibration to starting conditions over 2 min. Flow rates and column temperature were kept constant at 0.4 mL min^−1^ and 25 °C, respectively. UV absorbance was detected at 254 nm and 280 nm throughout. HPLC retention time of peptides were assigned based on mass extraction.

Purification of the peptides was performed by Agilent 1260 Infinity II LC system. 900 μL of solution containing crude mixture dissolved in H_2_O/MeCN was injected onto a Phenomenex Jupiter semi-preparative C12 HPLC column (4 μ packing, 250 × 10 mm). Starting conditions of 50% (*v*/*v*) MeCN/H_2_O (plus 0.1 % (*v*/*v*) TFA) were held for 2 min prior to development to 90% (*v*/*v*) MeCN/H_2_O over 15 min 95% (*v*/*v*) MeCN/H_2_O, then held for 3 min prior to re-equilibration of starting conditions over 3 min. Flow rates were kept constant at 5 mL min^−1^. UV absorbance was detected at 280 nm throughout.

## 3. Results

The TH enzymes PyrH, SttH, ThHal, PrnA and RebH were first assayed for chlorination of a panel of short (2–4 mer) peptides comprising only tryptophan (W) and glycine (G) residues. PyrH, SttH and ThHal chlorinated peptides with tryptophan at the C-terminus (Table 1). The enzymes PrnA and RebH that halogenate at the tryptophan 7 position did not produce any detectable chlorinated products. Interestingly, only mono-chlorinated products were observed, even for the peptides containing two tryptophan residues. Chlorination of WW and GW dipeptides was observed but not WG, suggesting that only the C-terminal tryptophan residue was accessible to the substrate binding site.

**Figure 1:**
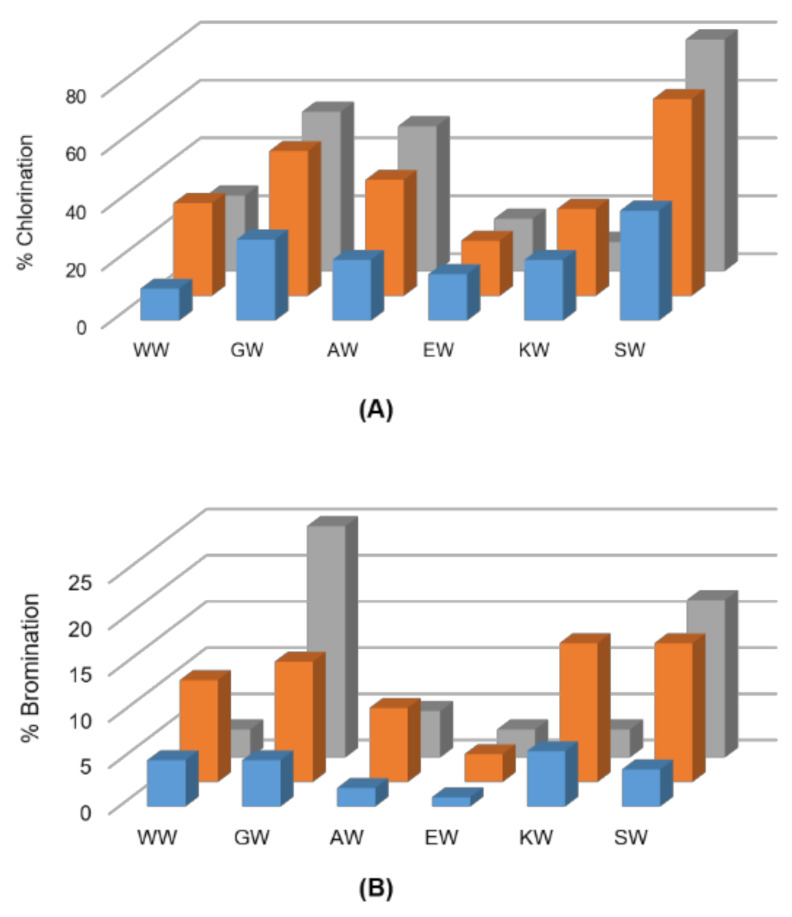
Screen for (**A**) chlorination and (**B**) bromination of indicated dipeptides using ThHal (blue), SttH (orange) and PyrH (grey) enzymes.

Next, we expanded the panel of screened dipeptide substrates to include charged, nucleophilic and bulkier amino acid residues adjacent to the C-terminus tryptophan. PyrH exhibited very high activity on SW, GW and AW dipeptides, showing 83%, 55% and 50% chlorination, respectively (Figure 1). SttH and ThHal also showed higher activity on these peptides. Peptides with a bulkier or charged amino acid next to the C-terminal tryptophan showed reduced halogenation. All three enzymes were able to brominate the dipeptides, although with lower efficiencies. The most efficient enzyme PyrH was able to brominate 25% of the GW and 17% of the SW peptide.

**Figure 2:**
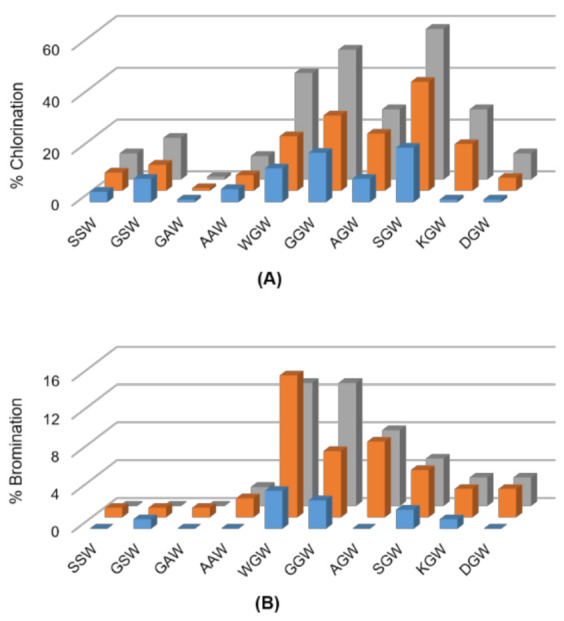
Screen for (**A**) chlorination and (**B**) bromination of indicated tripeptides using ThHal (blue), SttH (orange) and PyrH (grey) enzymes.

Tripeptides comprising the permissive SW, GW and AW motifs extended by either a bulky, nucleophilic, or charged amino acid at the N-terminus were next investigated. The halogenases were largely inefficient at chlorinating the tripeptides SSW, GSW, AAW or GAW, and almost inactive for their bromination (Figure 2). PyrH-catalyzed chlorination dropped from 83% for SW dipeptide to 16% and 10% for GSW and SSW tripeptides, respectively. Activity on the AAW (9%) and GAW (1%) tripeptides was also markedly lower than for the AW dipeptide (50%). In contrast, chlorination and bromination of the tripeptide GGW (50%) was comparable to GW dipeptide (55%). Highest activity was seen for the SGW tripeptide, with 58% chlorination achieved by PyrH. Incorporation of charged amino acids reduced efficiency in all sequence contexts.

**Figure 3:**
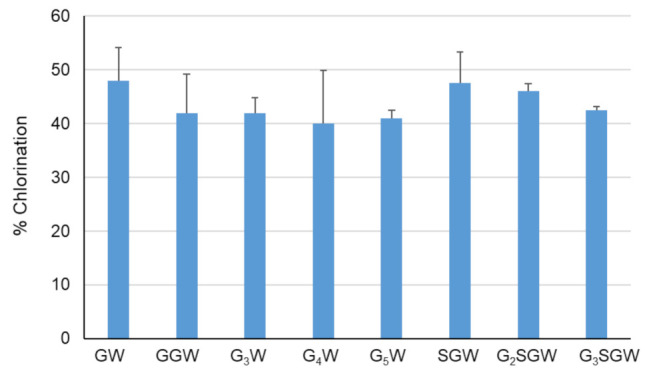
Chlorination of indicated peptides using PyrH. Values represent average ± SD (*n* = 2).

The effect of peptide length on PyrH-catalyzed chlorination was tested next by sequential N-terminal extension of the GGW and SGW tripeptides with glycine (Figure 3). All peptides were efficiently converted in comparison to the GW dipeptide and GGW /SGW tripeptides. The longer G_5_W and G_3_SGW peptides were next enzymatically chlorinated and purified at preparative scale. ^1^H NMR spectra indicated tryptophan C5 as the sole halogenation site (Figures S1–S4, S6 and S7, in agreement with the regiospecificity of PyrH [25]. To further confirm halogenation, Suzuki coupling of 1,4-benzenediboronic acid bis(pinacol) ester to the PyrH-chlorinated G_3_SGW peptide was carried out. Phenyl-substituted G_3_SGW peptide formed by monocoupling and subsequent protodeboronation was detected by LC-MS (Figure S35). This indicates the possibility of derivatization of enzymatically chlorinated peptides, although further protocol optimization is required to achieve efficient dicoupling.

**Figure 4:**
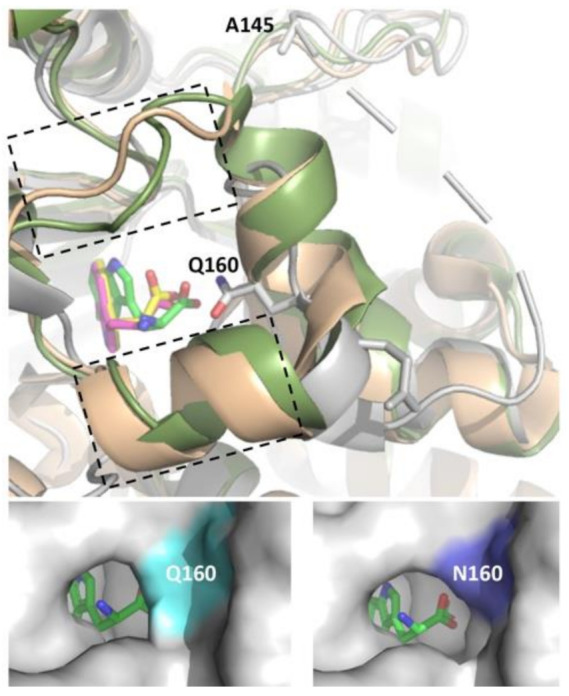
Top Panel: Structural overlay of PyrH (grey, bound tryptophan in green), PrnA (wheat, bound tryptophan in magenta) and RebH (green, bound tryptophan in yellow). Boxed region denotes α-helical region (F458–N464 in RebH and F447–N453 in PrnA) and loop region (T343–F438 in RebH and N444–S448 in PrnA) that is absent in PyrH. The PyrH residues Q160 (mutated to N in Q160N variant) and A145 (deleted in PyrH–dASQV) are highlighted. Note that S146, Q147 and V148 also deleted in this mutant are not resolved in crystal structure. Bottom panels: Modeling of the PyrH Q160N mutation with bound tryptophan highlights cavitation of the active site region enabling access to larger peptidic substrates. Images generated based on structures 2WEU [26] (PyrH), 2OA1 [30] (RebH) and 2ARD [29] (PrnA) using PyMOL.

**Figure 5:**
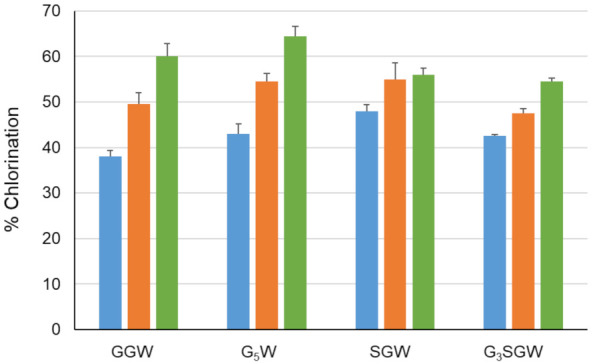
Peptide chlorination using WT PyrH (blue), PyrH-dASQV (orange) and PyrH-Q160N (green) for indicated peptides. Values represent average ± SD (*n* = 2).

The PyrH substrate binding pocket differs notably from PrnA and RebH, resulting in tryptophan adopting a different bound conformation [26]. Differences include both an α-helical region in the T7H enzymes (F458–N464 in RebH and F447–N453 in PrnA) and a short loop insertion (T343–F438 in RebH and N444–S448 in PrnA) that potentially limits optimal peptide binding by active site occlusion. No similar structural elements are present in PyrH, likely rendering its active site more accessible to the larger non-cognate peptide substrates (Figure 4). We hypothesized that further opening-up of the PyrH substrate binding site could enhance halogenation efficiency. We therefore deleted four amino acids (A145–V148) within a proximal unstructured loop region (F144–Y166) to yield variant PyrH-dASQV. This enzyme showed between 10 to 30% increased activity over the wild-type PyrH on peptide substrates (Figure 5). We also introduced a conservative mutation, Q160N, to both increase active site accessibility and further accommodate residues preceding the C-terminal tryptophan (Figure 4). This variant (PyrH-Q160N) showed ≥50% higher activity over PyrH for chlorination of GGW and G_5_W peptides (Figure 5). The binding affinity (K_M_) of PyrH-Q160N was 54% improved over PyrH for the GGW peptide (Table 2). Both variants exhibited similar turn-over number (kcat), indicating that the modest increase in activity of PyrH-Q160N arose from improved substrate binding.

**Figure 6:**
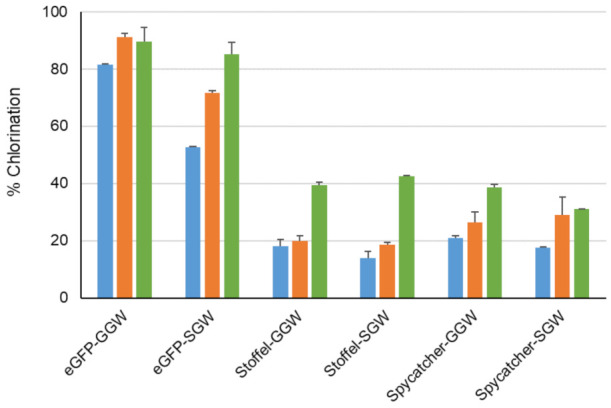
Chlorination of indicated proteins using WT PyrH (blue), PyrH-dASQV (orange) and PyrH-Q160N (green) mutants. Values represent average ± SD (*n* = 2).

We next recombinantly expressed and purified three model proteins, eGFP [36] (30 KDa), Stoffel fragment [37] of Taq DNA polymerase (64 KDa) and SpyCatcher [38] (13 KDa) with genetically encoded C-terminal GGW or SGW tags preceded by a site-specific protease cleavage site to facilitate MS analysis of reaction products (Tables S1 and S2). C-terminal halogenation by PyrH and its two mutant variants was observed (Figure 6). This approached 90% conversion for the eGFP-GGW substrate using the PyrH mutants, with no significant perturbation of its spectral properties compared to unmodified eGFP (Figure S37). A control experiment utilizing the tagged thermostable Stoffel fragment and heat inactivation of the halogenase enzyme (65 °C, 20 min) prior to proteolysis and MS analysis confirmed protein halogenation (Figure S5). As before, PyrH-Q160N was generally the most active (Figure 4). Lower halogenation efficiencies of the Stoffel and SpyCatcher proteins (~40% using PyrH-Q160N) was observed, which may arise from steric hindrance between the halogenase and the substrate protein. Further iteration of linker lengths between these proteins and the (G/S)GW tag is warranted to address this possibility.

## 4. Discussion

We have developed a novel enzymatic method for in vitro C-terminal halogenation of a range of peptides and proteins. Enzyme and substrate screening yielded peptides comprising the optimal (G/S)GW motif, which we term the HaloTryp Tag. In combination with the rationally designed PyrH-Q160N mutant, the HaloTryp Tag facilitated site-specific halogenation of several model proteins (40 to 90% conversion). We also demonstrated chemo-catalytic derivatization of an enzymatically halogenated peptide, although cross-coupling was not observed with the protocol employed. In this respect, derivatization of halogenated peptides/proteins should be assessed using recently described cross-coupling approaches that work at low temperatures in aqueous media [39,40,41,42]. Post-translational labelling using the HaloTryp Tag could also be used downstream of co-translational labelling methodologies [18,43] to expand chemical diversity and potentially introduce novel physico-chemical properties. Of pertinent interest is modulation of a therapeutic protein’s cell permeability via halogen installation.

## Figures and Tables

**Table 1:** Chlorination of short peptides using five wild type halogenase enzymes.

Substrate	PyrH	SttH	ThHal	PrnA	RebH
WW	+	+	+	−	−
WG	−	−	−	−	−
GW	+	+	+	−	−
GWG	−	−	−	−	−
WGW	+	+	+	−	−
WWG	−	−	−	−	−
GWW	+	+	+	−	−
GGWG	−	−	−	−	−
GWGG	−	−	−	−	−

**Table 2:** Kinetic parameters for chlorination of GGW peptide using PyrH and PyrH-Q160N enzymes. Values indicate average ± SD (*n* = 2).

Enzyme	K_M_ (mM)	k_cat_ (min^−1^)	k_cat_/K_M_ (min^−1^ mM^−1^)
PyrH	2.77 ± 0.12	2.50 ± 0.09	0.90 ± 0.033
PyrH-Q160N	1.79± 0.11	2.52 ± 0.12	1.40 ± 0.052

## Data Availability

Not applicable.

## Supplementary Materials

The following supporting information can be downloaded at: https://www.mdpi.com/article/10.3390/biom12121841/s1, Figure S1. 1H Spectra of chlorinated GGGGGW; Figure S2. Chemical structure of chlorinated GGGGGW; Figure S3. 1H Spectra of chlorinated GGGSGW; Figure S4. Chemical structure of chlorinated GGGSGW; Figure S5. Chlorination of Stoffel-SGW protein using PyrH-Q160N enzyme followed by C-terminus cleavage with and without prior heat inactivation of the halogenating enzymes; Figure S6. HRMS of chlorinated GGGGGW; Figure S7. HRMS of chlo-rinated GGGSGW; Figure S8. HPLC spectrum for chlorination of eGFP-GGW produced by enzy-matic halogenation using WT PyrH. Retention time before and after chlorination are 7.210 min and 7.817 min, respectively; Figure S9. HPLC spectrum for chlorination of eGFP-GGW produced by enzymatic halogenation using PyrH-dASQV; Figure S10. HPLC spectrum for chlorination of eGFP-GGW produced by enzymatic halogenation using PyrH-Q160N. Retention time before and after chlorination are 6.005 min and 6.628 min, respectively; Figure S11. HPLC spectrum for chlorination of eGFP-SGW produced by enzymatic halogenation using WT PyrH; Figure S12. HPLC spectrum for chlorination of Egfp-SGW produced by enzymatic halogenation using PyrH-Dasqv; Figure S13. HPLC spectrum for chlorination of Egfp-SGW produced by enzymatic halogenation using PyrH-Q160N; Figure S14. HPLC spectrum for chlorination of Stoffel-GGW produced by enzymatic halogenation using WT PyrH; Figure S15. HPLC spectrum for chlorination of Stoffel-GGW produced by enzymatic halogenation using PyrH-Dasqv; Figure S16. HPLC spec-trum for chlorination of Stoffel-GGW produced by enzymatic halogenation using PyrH-Q160N; Figure S17. HPLC spectrum for chlorination of Stoffel-SGW produced by enzymatic halogenation using WT PyrH; Figure S18. HPLC spectrum for chlorination of Stoffel-SGW produced by enzy-matic halogenation using PyrH-dASQV; Figure S19. HPLC spectrum for chlorination of Stof-fel-SGW produced by enzymatic halogenation using PyrH-Q160N; Figure S20. HPLC spectrum for chlorination of Spycatcher-GGW produced by enzymatic halogenation using WT PyrH. Retention time before and after chlorination are 7.205 min and 7.859 min, respectively; Figure S21. HPLC spectrum for chlorination of Spycatcher-GGW produced by enzymatic halogenation using PyrH-dASQV. Retention time before and after chlorination are 7.2222 min and 7.861 min, respec-tively; Figure S22. HPLC spectrum for chlorination of Spycatcher-GGW produced by enzymatic halogenation using PyrH-Q160N. Retention time before and after chlorination are 6.936 min and 7.555 min, respectively; Figure S23. HPLC spectrum for chlorination of Spycatcher-SGW produced by enzymatic halogenation using WT PyrH. Retention time before and after chlorination are 7.137 min and 7.791 min, respectively; Figure S24. HPLC spectrum for chlorination of Spycatcher-SGW produced by enzymatic halogenation using PyrH-dASQV. Retention time before and after chlo-rination are 7.150 min and 7.794 min, respectively. No di and tri substituted product were observed by mass extraction in MS; Figure S25. HPLC spectrum for chlorination of Spycatcher-SGW pro-duced by enzymatic halogenation using PyrH-Q160N. Retention time before and after chlorination are 6.888 min and 7.510 min, respectively; Figure S26. HPLC spectrum for chlorination of GW produced by enzymatic halogenation using PyrH; Figure S27. HPLC spectrum for chlorination of G2W produced by enzymatic halogenation using PyrH; Figure S28. HPLC spectrum for chlorina-tion of G3W produced by enzymatic halogenation using PyrH. Retention time before and after chlorination are 1.788 min and 2.211 min, respectively; Figure S29. HPLC spectrum for chlorina-tion of G4W produced by enzymatic halogenation using PyrH; Figure S30. HPLC spectrum for chlorination of G5W produced by enzymatic halogenation using PyrH; Figure S31. HPLC spectrum for chlorination of SGW produced by enzymatic halogenation using PyrH; Figure S32. HPLC spectrum for chlorination of G2SGW produced by enzymatic halogenation using PyrH; Figure S33. HPLC spectrum for chlorination of G3SGW produced by enzymatic halogenation using PyrH; Figure S34. Michaelis-Menten plot for calculating kinetic parameters for chlorination of GGW using WT PyrH (solid circles) and PyrH-Q160N (solid squares). Values represent average ±SD (n = 2); Figure S35. Extracted MS (ESI+) spectrum of phenyl substituted G3SGW; Figure S36. SDS-PAGE analysis of indicated purified proteins; Figure S37. A. Absorbance spectra of eGFP and eGFP hal-ogenated with PyrH-Q160N enzyme (5 mg/mL) in 10 mM phosphate buffer (pH = 7.2); Table S1. Amino acid sequence of proteins tested for halogenation showing the N-terminus His-tag in orange, the protein in green, PreScission protease cleavage sequence in blue, FLAG (solubility) tag in red and the HaloTrypTag in black; Table S2. Primers used for inserting LEVLFQGPDYKDDDDK-GGW/-SGW sequence at the C-terminus of respective proteins.
