# Supplementary material for: Halogenation of Peptides and Proteins Using Engineered Tryptophan Halogenase Enzymes
Source: Biomolecules. 2022 Dec 8;12(12):1841. doi: 10.3390/biom12121841 (PMC9775415; doi:10.3390/biom12121841)
Supplement: Supplementary file 1 [file biomolecules-12-01841-s001.zip › biomolecules-2027702-supplementary.pdf]

SUPPLEMENTARY TABLES

Table S1. Amino acid sequence of proteins tested for halogenation showing the N-terminus His-tag in orange, the protein in green, PreScission protease cleavage sequence in blue, FLAG (solubility) tag in red and the HaloTrypTag in black.

Protein	Sequence	Molecular weight (kD)
GFP-GGW	MHHHHHHMVSKGEELFTGVVPILVELDGDVNGHKFSVSGEGEGDATYGKLTLKF<br>ICTTGKLPVPWPTLVTTLTYGVQCFSRYPDHMKQHDFFKSAMPEGYVQERTIFF<br>KDDGNYKTRAEVKFEGDTLVNRIELKGIDFKEDGNILGHKLEYNYNSHNVYIMA<br>DKQKNGIKVNFKIRHNIEDGSVQLADHYQQNTPIGDGPVLLPDNHYLSTQSALS<br>KDPNEKRDHMVLLEFVTAAGITLGMDELYKLEVLFQGPDYKDDDDKGGW	30.07
GFP-SGW	MHHHHHHMVSKGEELFTGVVPILVELDGDVNGHKFSVSGEGEGDATYGKLTLKF<br>ICTTGKLPVPWPTLVTTLTYGVQCFSRYPDHMKQHDFFKSAMPEGYVQERTIFF<br>KDDGNYKTRAEVKFEGDTLVNRIELKGIDFKEDGNILGHKLEYNYNSHNVYIMA<br>DKQKNGIKVNFKIRHNIEDGSVQLADHYQQNTPIGDGPVLLPDNHYLSTQSALS<br>KDPNEKRDHMVLLEFVTAAGITLGMDELYKLEVLFQGPDYKDDDDKSGW	30.10
Stoffel-GGW	MHHHHHHEAPWPPPEGAFVGFVLSRKEPMWADLLALAAARGGRVHRAPEPYKAL<br>RDLKEARGLLAKDLSVLALREGLGLPPGDDPMLLAYLLDPSNTTPEGVARRYGG<br>EWTEEAGERAALSERLFANLWGRLEGEERLLWLYREVERPLSAVLAHMEATGVR<br>LDVAYLRALSLEVAEEIARLEAEVFRLAGHPFNLNSRDQLERVLFDELGLPAIG<br>KTEKTGKRSTSAAVLEALREAHPIVEKILQYRELTKLKSTYIDPLPDLIHPRTG<br>RLHTRFNQTATATGRLSSSDPNLQNIPVRTPLGQRIRRAFIAEEGWLLVALDYS<br>QIELRVLAHLSGDENLIRVFQEGRDIHTETASWMFGVPREAVDPLMRRAAKTIN<br>FGVLYGMSAHRLSQELAIPYEEAQAFIERYFQSFPKVRAWIEKTLEEGRRRGYV<br>ETLFGRRRYVPDLEARVKSVREAAERMAFNMPVQGTAADLMKLAMVKLFPRLEE<br>MGARMLLQVHDELVLEAPKERAEAVARLAKEVMEGVYPLAVPLEVEVGIGEDWL<br>SAKELEVLFQGPDYKDDDDKGGW	63.67
Stoffel-SGW	MHHHHHHEAPWPPPEGAFVGFVLSRKEPMWADLLALAAARGGRVHRAPEPYKAL<br>RDLKEARGLLAKDLSVLALREGLGLPPGDDPMLLAYLLDPSNTTPEGVARRYGG<br>EWTEEAGERAALSERLFANLWGRLEGEERLLWLYREVERPLSAVLAHMEATGVR<br>LDVAYLRALSLEVAEEIARLEAEVFRLAGHPFNLNSRDQLERVLFDELGLPAIG<br>KTEKTGKRSTSAAVLEALREAHPIVEKILQYRELTKLKSTYIDPLPDLIHPRTG<br>RLHTRFNQTATATGRLSSSDPNLQNIPVRTPLGQRIRRAFIAEEGWLLVALDYS<br>QIELRVLAHLSGDENLIRVFQEGRDIHTETASWMFGVPREAVDPLMRRAAKTIN<br>FGVLYGMSAHRLSQELAIPYEEAQAFIERYFQSFPKVRAWIEKTLEEGRRRGYV<br>ETLFGRRRYVPDLEARVKSVREAAERMAFNMPVQGTAADLMKLAMVKLFPRLEE<br>MGARMLLQVHDELVLEAPKERAEAVARLAKEVMEGVYPLAVPLEVEVGIGEDWL<br>SAKELEVLFQGPDYKDDDDKSGW	63.70
Spycatcher-GGW	MHHHHHHEMDSATHIKFSKRDEDGKELAGATMELRDSSGKTISTWISDGQVKDF<br>YLYPGKYTFVETAAPDGYEVATAITFTVNEQGQVTVNGKATKGDAHLEVLFQGP<br>DYKDDDDKGGW	13.26
Spycatcher-SGW	MHHHHHHEMDSATHIKFSKRDEDGKELAGATMELRDSSGKTISTWISDGQVKDF<br>YLYPGKYTFVETAAPDGYEVATAITFTVNEQGQVTVNGKATKGDAHLEVLFQGP<br>DYKDDDDKSGW	13.29

Table S2. Primers used for inserting LEVLFQGPDYKDDDDK-GGW/-SGW sequence at the C-terminus of respective proteins.

Protein	Primer	Sequence
GFP-GGW	Forward	5'- GAT TAC AAA GAC GAC GAT GAT AAA GGT GGT TGG TAA GGA TCC GAA TTC GAG CTC CGT -3'
	Reverse	5'- AGG TCC TTG AAA TAA AAC TTC CAG CTT GTA CAG CTC GTC CAT GCC GAG AGT GA -3'
GFP-SGW	Forward	5'- GAT TAC AAA GAC GAC GAT GAT AAA TCT GGT TGG TAA GGA TCC GAA TTC GAG CTC CGT -3'
	Reverse	5'- AGG TCC TTG AAA TAA AAC TTC CAG CTT GTA CAG CTC GTC CAT GCC GAG AGT GA -3'
Stoffel-GGW	Forward	5'- GAT TAC AAA GAC GAC GAT GAT AAA GGT GGT TGG TAA GGA TCC GAA TTC GAG CTC CGT -3'
	Reverse	5'- AGG TCC TTG AAA TAA AAC TTC CAG TTC CTT GGC ACT CAG CCA ATC TTC GCC A -3'
Stoffel-SGW	Forward	5'- GAT TAC AAA GAC GAC GAT GAT AAA TCT GGT TGG TAA GGA TCC GAA TTC GAG CTC CGT -3'
	Reverse	5'- AGG TCC TTG AAA TAA AAC TTC CAG TTC CTT GGC ACT CAG CCA ATC TTC GCC A -3'
Spycatcher-GGW	Forward	5'- GAT TAC AAA GAC GAC GAT GAT AAA GGT GGT TGG TAA GGA TCC GAA TTC GAG CTC CGT -3'
	Reverse	5'- AGG TCC TTG AAA TAA AAC TTC CAG ATG TGC ATC ACC CTT GGT GGC TTT GC -3'
Spycatcher-SGW	Forward	5'- GAT TAC AAA GAC GAC GAT GAT AAA TCT GGT TGG TAA GGA TCC GAA TTC GAG CTC CGT -3'
	Reverse	5'- AGG TCC TTG AAA TAA AAC TTC CAG ATG TGC ATC ACC CTT GGT GGC TTT GC -3'

SUPPLEMENTARY FIGURES

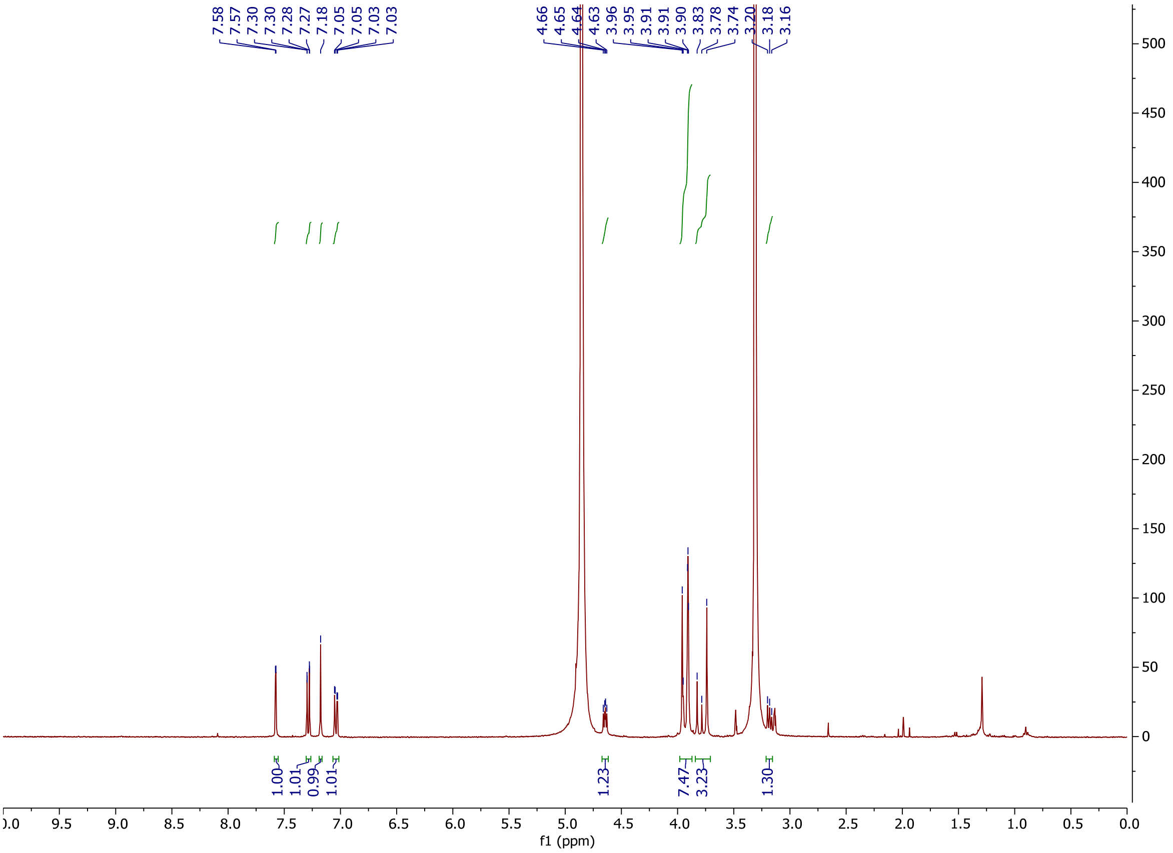

Figure S1. [1]H Spectra of chlorinated GGGGGW.

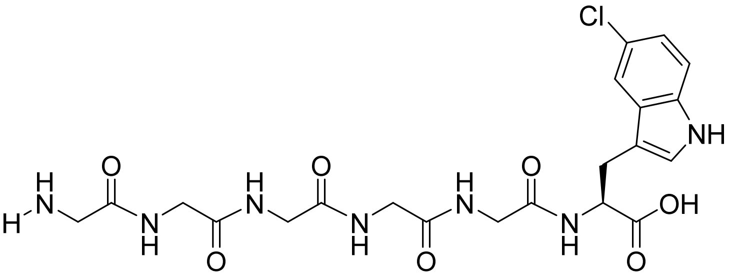

Figure S2. Chemical structure of chlorinated GGGGGW.

Isolated yield: 1.0 mg (19%).

1H NMR (400 MHz, CD_3OD): δ 7.58 (d, J = 1.7 Hz, 1H), 7.29 (dd, J = 8.6, 0.5 Hz, 1H), 7.18 (s, 1H), 7.04 (dd, J = 8.6, 2.1 Hz, 1H), 4.64 (dd, J = 7.7, 5.1 Hz, 1H), 3.99 – 3.87 (m, 7H), 3.84 – 3.70 (m, 3H), 3.21 – 3.16 (m, 1H).

HRMS (ESI+): m/z calcd for $C_{21}H_{26}ClN_7O_7$ $[M+H]^+$ 524.1655, found 524.1655.

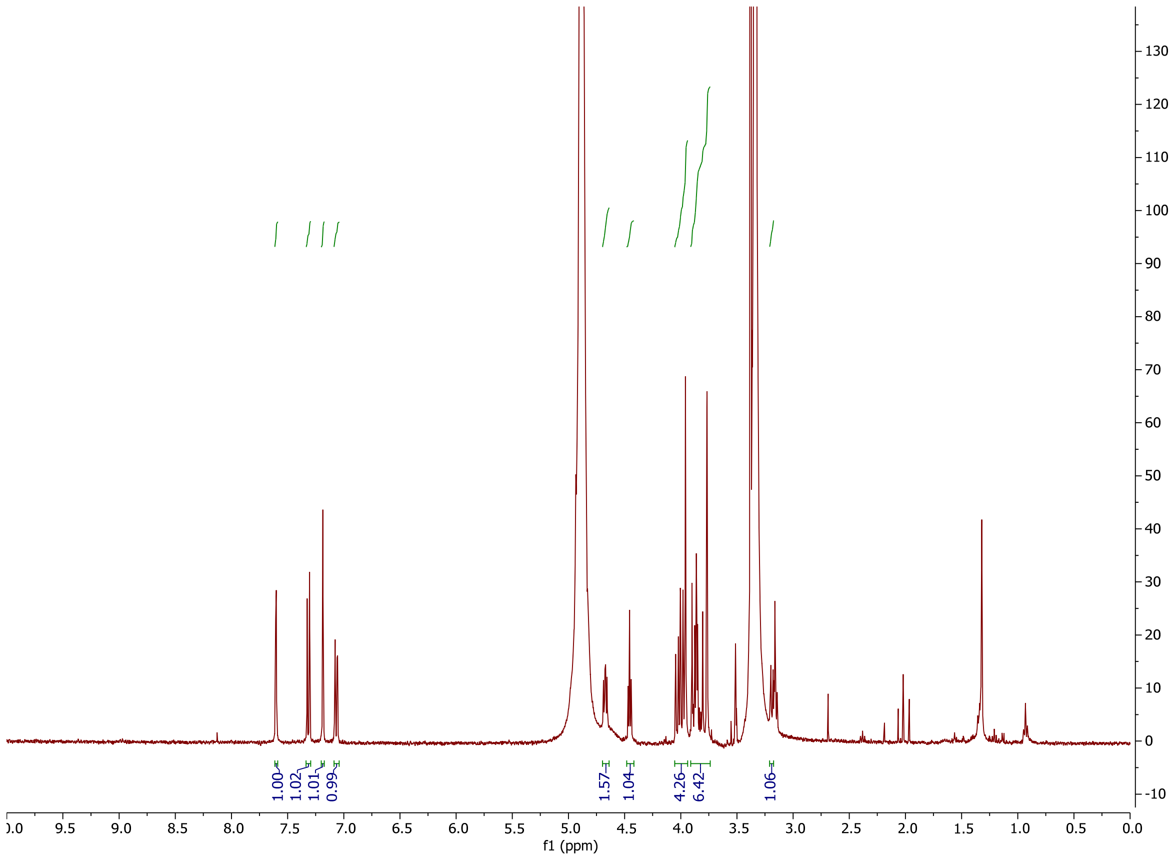

Figure S3. ^{1}H Spectra of chlorinated GGGSGW.

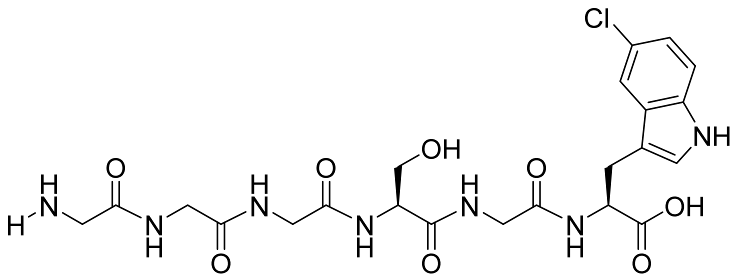

Figure S4. Chemical structure of chlorinated GGGSGW.

Isolated yield: 1.7 mg (32%).

1H NMR (400 MHz, CD_3OD): δ 7.60 (dd, J = 2.1, 0.6 Hz, 1H), 7.31 (dd, J = 8.6, 0.6 Hz, 1H), 7.19 (s, 1H), 7.07 (dd, J = 8.6, 2.0 Hz, 1H), 4.67 (dd, J = 7.8, 5.0 Hz, 1H), 4.46 (t, J = 5.3 Hz, 1H), 4.05 – 3.93 (m, 4H), 3.91 – 3.74 (m, 6H), 3.21 – 3.13 (m, 1H).

HRMS (ESI+): m/z calcd for $C_{22}H_{28}ClN_7O_8$ $[M+H]^+$ 554.1761, found 554.1767.

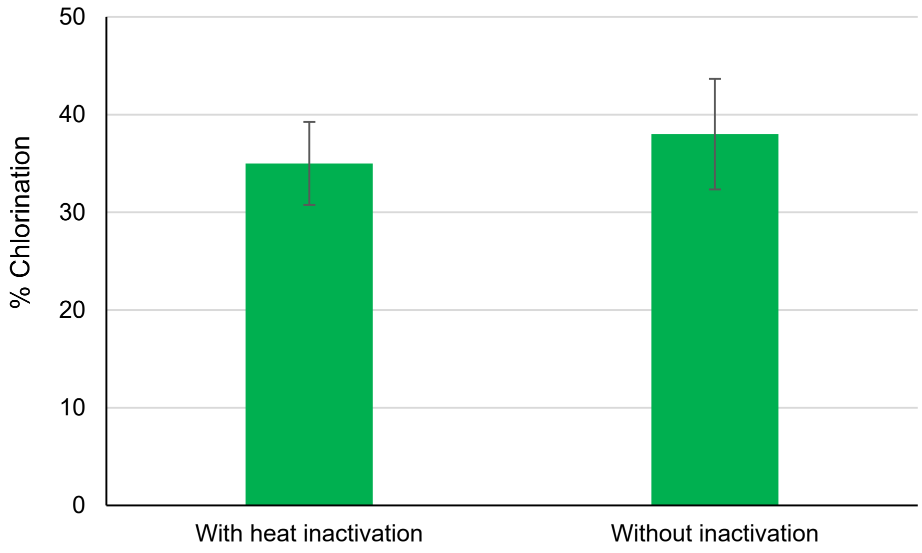

Figure S5. Chlorination of Stoffel-SGW protein using PyrH-Q160N enzyme followed by C-terminus cleavage with and without prior heat inactivation of the halogenating enzymes. Values represent average ± SD (n=2).

SUPPORTING CHROMATOGRAMS

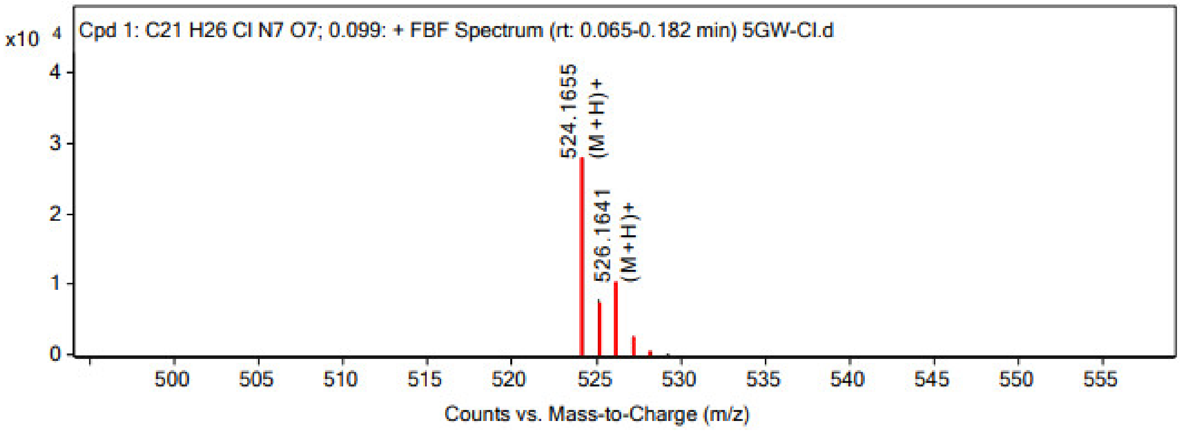

Figure S6. HRMS of chlorinated GGGGGW.

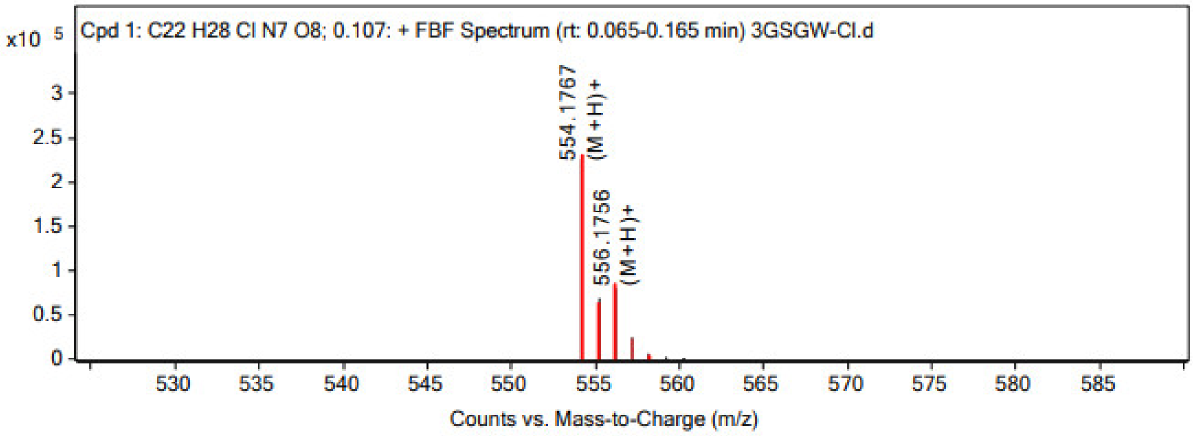

Figure S7. HRMS of chlorinated GGGSGW.

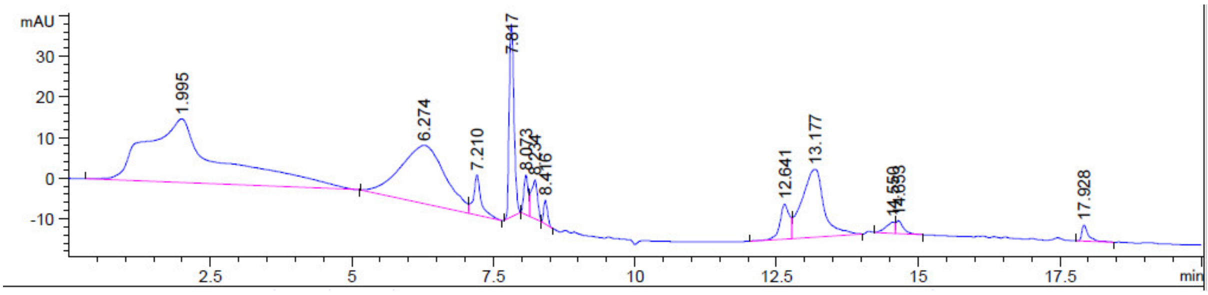

Figure S8. HPLC spectrum for chlorination of eGFP-GGW produced by enzymatic halogenation using WT PyrH. Retention time before and after chlorination are 7.210 min and 7.817 min, respectively. No di and tri substituted product were observed by mass extraction in MS.

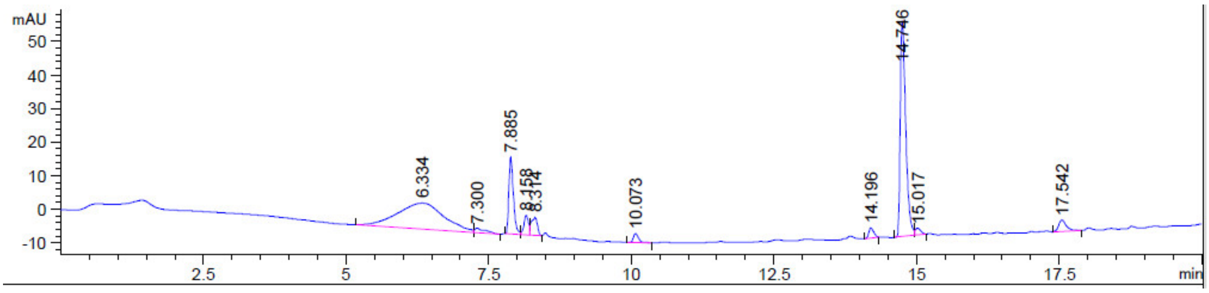

Figure S9. HPLC spectrum for chlorination of eGFP-GGW produced by enzymatic halogenation using PyrH-dASQV. Retention time before and after chlorination are 7.300 min and 7.885 min, respectively. No di and tri substituted product were observed by mass extraction in MS.

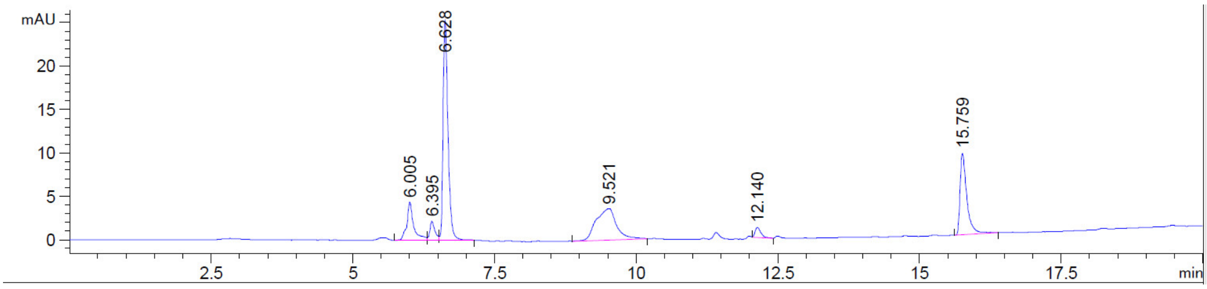

Figure S10. HPLC spectrum for chlorination of eGFP-GGW produced by enzymatic halogenation using PyrH-Q160N. Retention time before and after chlorination are 6.005 min and 6.628 min, respectively. No di and tri substituted product were observed by mass extraction in MS.

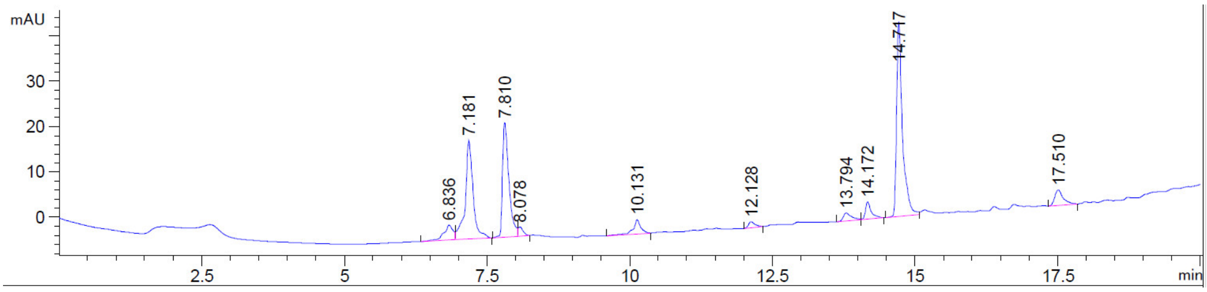

Figure S11. HPLC spectrum for chlorination of eGFP-SGW produced by enzymatic halogenation using WT PyrH. Retention time before and after chlorination are 7.181 min and 7.810 min, respectively. No di and tri substituted product were observed by mass extraction in MS.

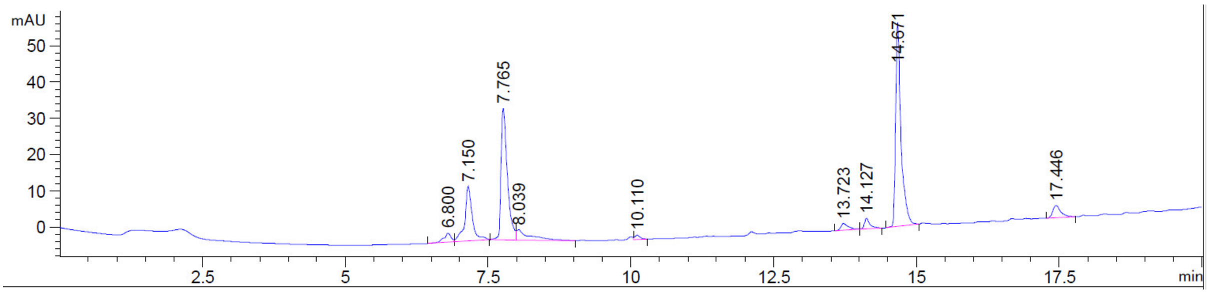

Figure S12. HPLC spectrum for chlorination of Egfp-SGW produced by enzymatic halogenation using PyrH-Dasqv. Retention time before and after chlorination are 7.150 min and 7.765 min respectively. No di and tri substituted product were observed by mass extraction in MS.

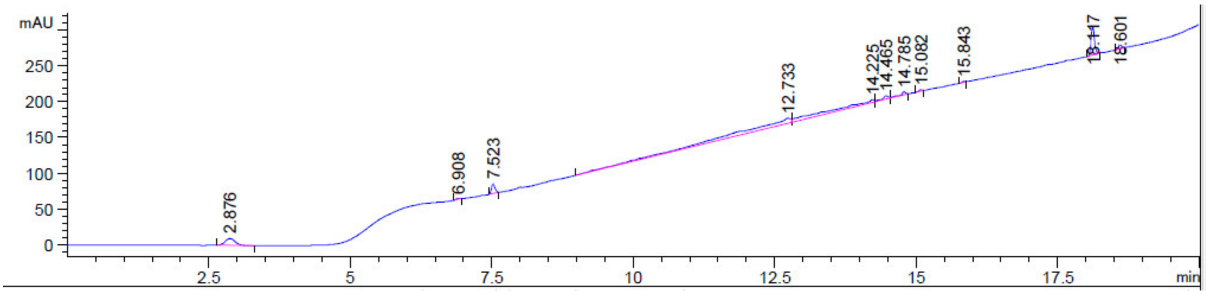

Figure S13. HPLC spectrum for chlorination of Egfp-SGW produced by enzymatic halogenation using PyrH-Q160N. Retention time before and after chlorination are 6.908 min and 7.523 min respectively. No di and tri substituted product were observed by mass extraction in MS.

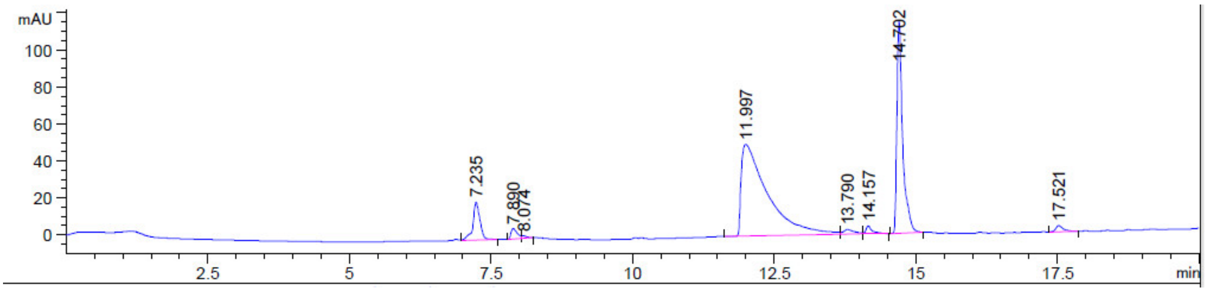

Figure S14. HPLC spectrum for chlorination of Stoffel-GGW produced by enzymatic halogenation using WT PyrH. Retention time before and after chlorination are 7.235 min and 7.890 min respectively. No di and tri substituted product were observed by mass extraction in MS.

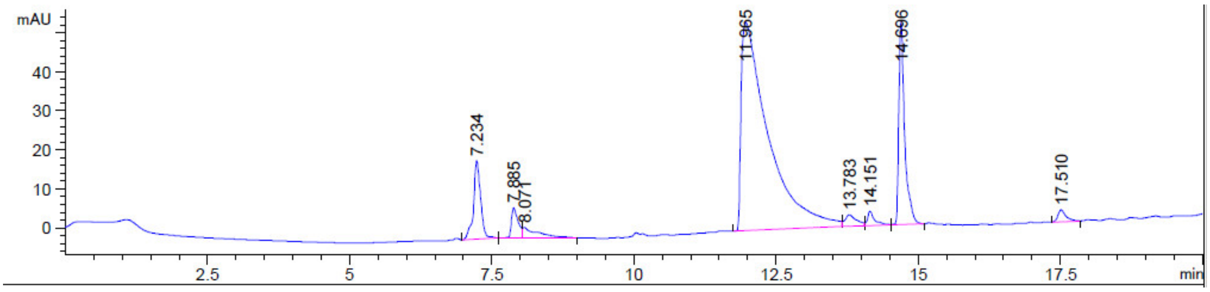

Figure S15. HPLC spectrum for chlorination of Stoffel-GGW produced by enzymatic halogenation using PyrH-Dasqv. Retention time before and after chlorination are 7.234 min and 7.885 min, respectively. No di and tri substituted product were observed by mass extraction in MS.

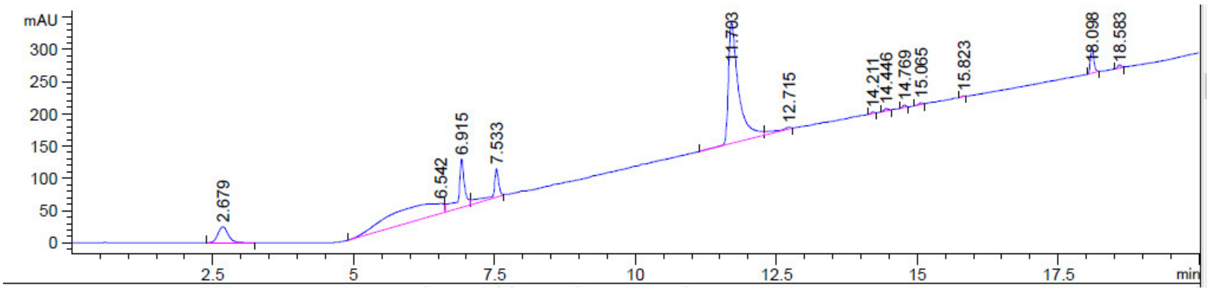

Figure S16. HPLC spectrum for chlorination of Stoffel-GGW produced by enzymatic halogenation using PyrH-Q160N. Retention time before and after chlorination are 6.915 min and 7.533 min respectively. No di and tri substituted product were observed by mass extraction in MS.

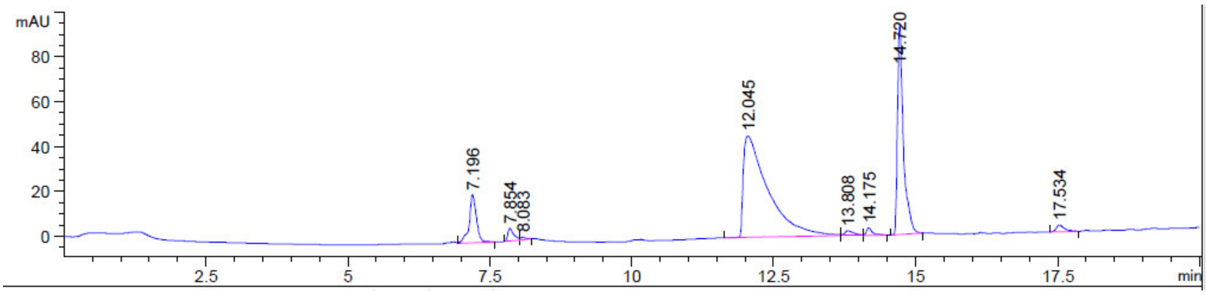

Figure S17. HPLC spectrum for chlorination of Stoffel-SGW produced by enzymatic halogenation using WT PyrH. Retention time before and after chlorination are 7.196 min and 7.854 min, respectively. No di and tri substituted product were observed by mass extraction in MS.

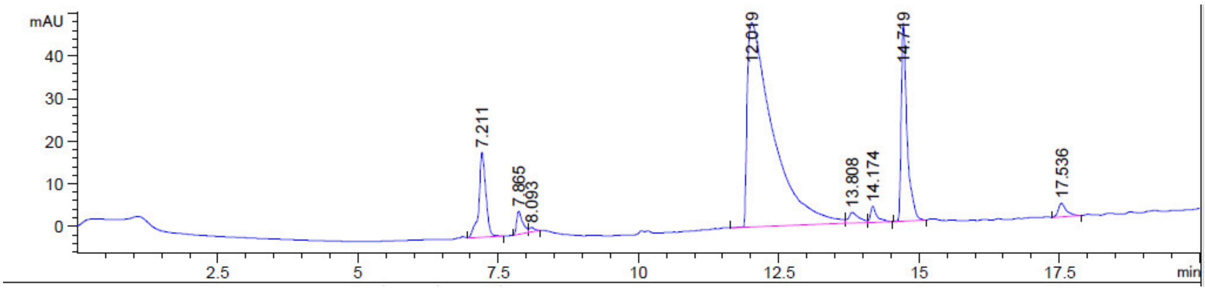

Figure S18. HPLC spectrum for chlorination of Stoffel-SGW produced by enzymatic halogenation using PyrH-dASQV. Retention time before and after chlorination are 7.211 min and 7.865 min, respectively. No di and tri substituted product were observed by mass extraction in MS.

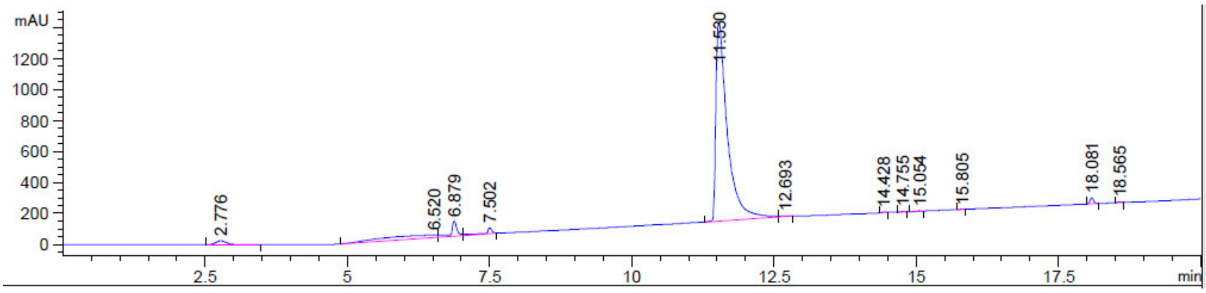

Figure S19. HPLC spectrum for chlorination of Stoffel-SGW produced by enzymatic halogenation using PyrH-Q160N. Retention time before and after chlorination are 6.879 min and 7.502 min, respectively. No di and tri substituted product were observed by mass extraction in MS.

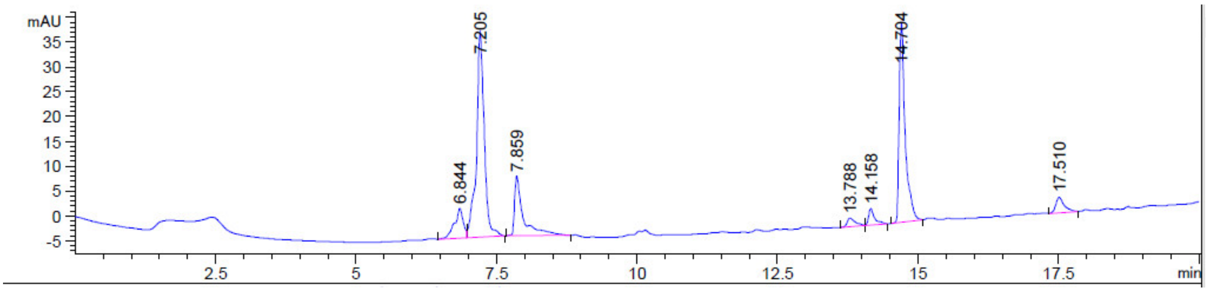

Figure S20. HPLC spectrum for chlorination of Spycatcher-GGW produced by enzymatic halogenation using WT PyrH. Retention time before and after chlorination are 7.205 min and 7.859 min, respectively. No di and tri substituted product were observed by mass extraction in MS.

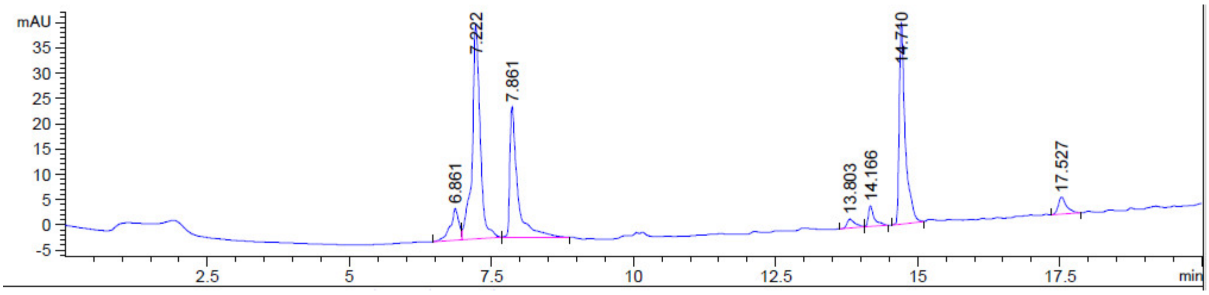

Figure S21. HPLC spectrum for chlorination of Spycatcher-GGW produced by enzymatic halogenation using PyrH-dASQV. Retention time before and after chlorination are 7.2222 min and 7.861 min, respectively. No di and tri substituted product were observed by mass extraction in MS.

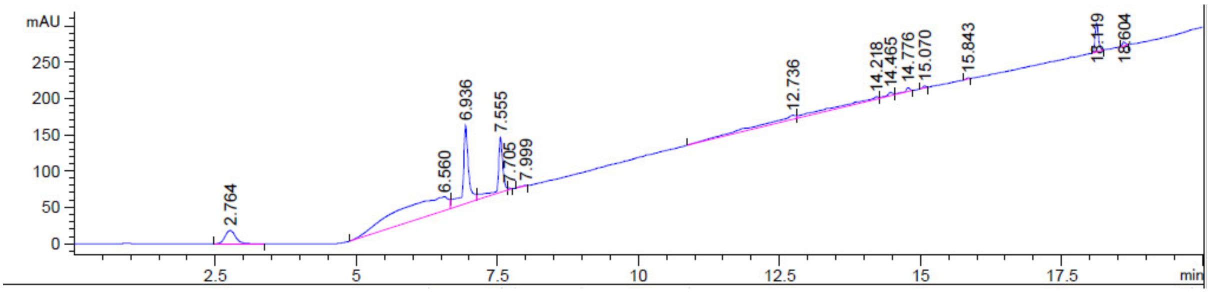

Figure S22. HPLC spectrum for chlorination of Spycatcher-GGW produced by enzymatic halogenation using PyrH-Q160N. Retention time before and after chlorination are 6.936 min and 7.555 min, respectively. No di and tri substituted product were observed by mass extraction in MS.

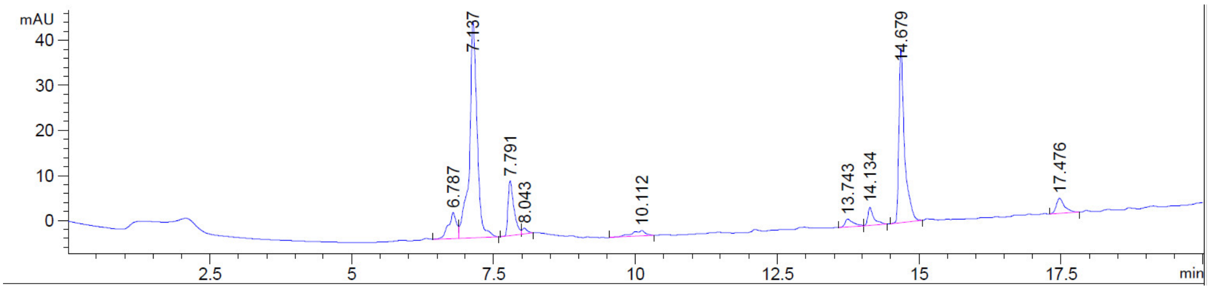

Figure S23. HPLC spectrum for chlorination of Spycatcher-SGW produced by enzymatic halogenation using WT PyrH. Retention time before and after chlorination are 7.137 min and 7.791 min, respectively. No di and tri substituted product were observed by mass extraction in MS.

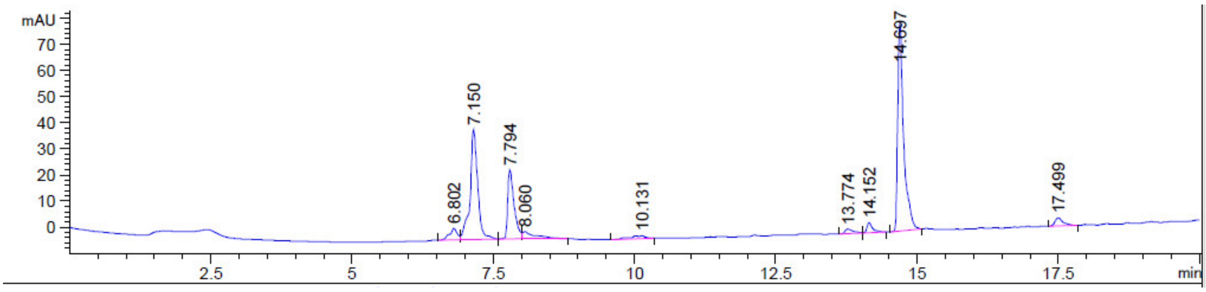

Figure S24. HPLC spectrum for chlorination of Spycatcher-SGW produced by enzymatic halogenation using PyrH-dASQV. Retention time before and after chlorination are 7.150 min and 7.794 min, respectively. No di and tri substituted product were observed by mass extraction in MS.

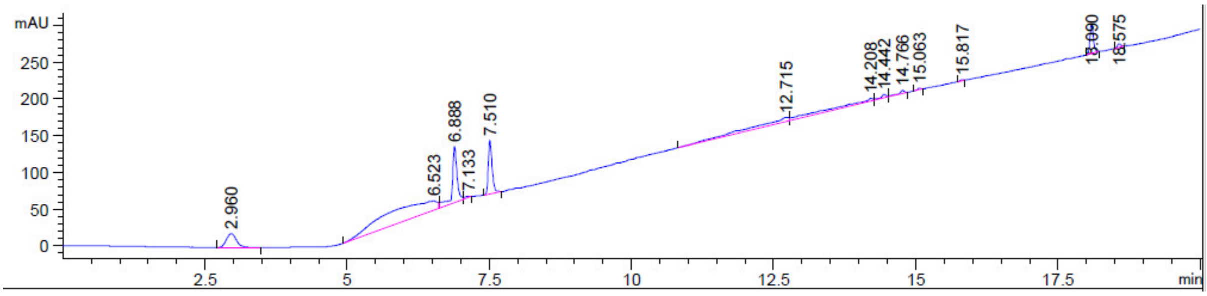

Figure S25. HPLC spectrum for chlorination of Spycatcher-SGW produced by enzymatic halogenation using PyrH-Q160N. Retention time before and after chlorination are 6.888 min and 7.510 min, respectively. No di and tri substituted product were observed by mass extraction in MS.

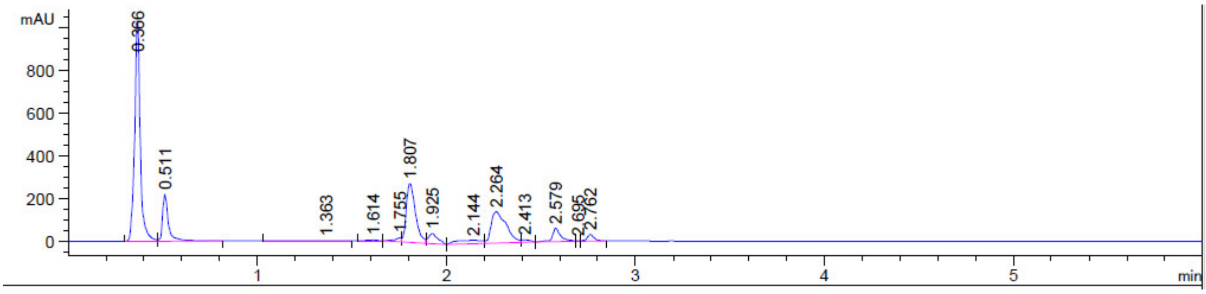

Figure 26. HPLC spectrum for chlorination of GW produced by enzymatic halogenation using PyrH. Retention time before and after chlorination are 1.807 min and 2.264 min, respectively. No di and tri substituted product were observed by mass extraction in MS.

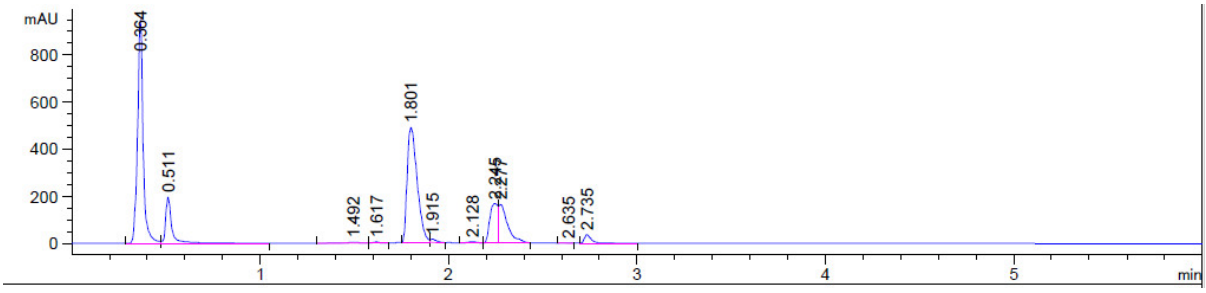

Figure S27. HPLC spectrum for chlorination of G_2W produced by enzymatic halogenation using PyrH. Retention time before and after chlorination are 1.801 min and 2.245 min, respectively. No di and tri substituted product were observed by mass extraction in MS.

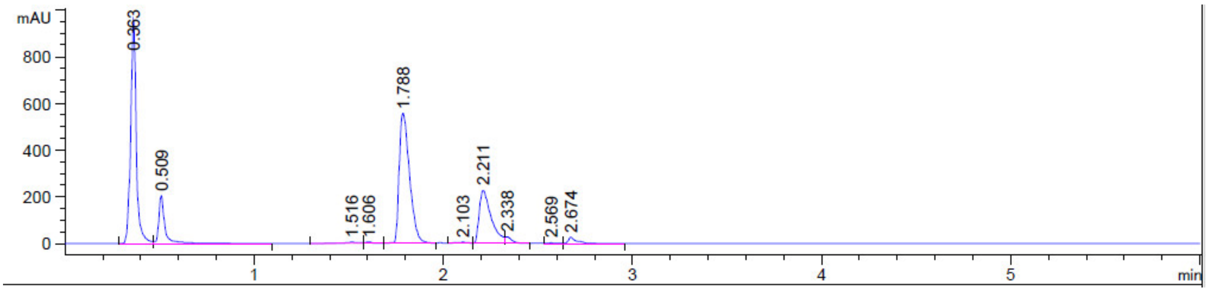

Figure S28. HPLC spectrum for chlorination of G_3W produced by enzymatic halogenation using PyrH. Retention time before and after chlorination are 1.788 min and 2.211 min, respectively. No di and tri substituted product were observed by mass extraction in MS.

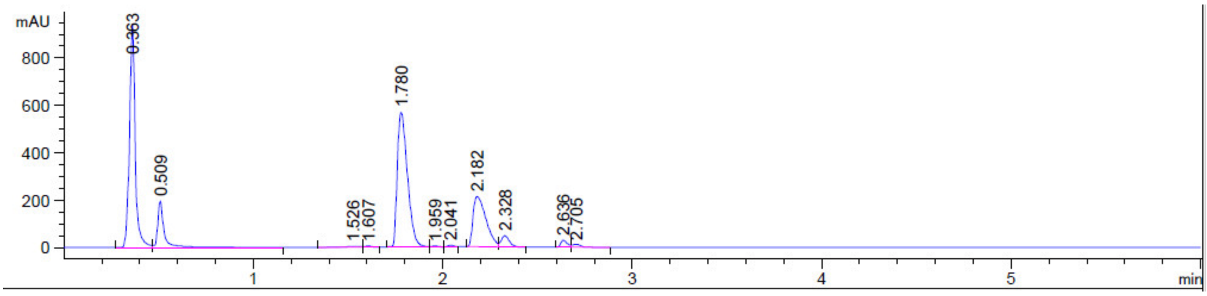

Figure S29. HPLC spectrum for chlorination of G_4W produced by enzymatic halogenation using PyrH. Retention time before and after chlorination are 1.780 min and 2.182 min, respectively. No di and tri substituted product were observed by mass extraction in MS.

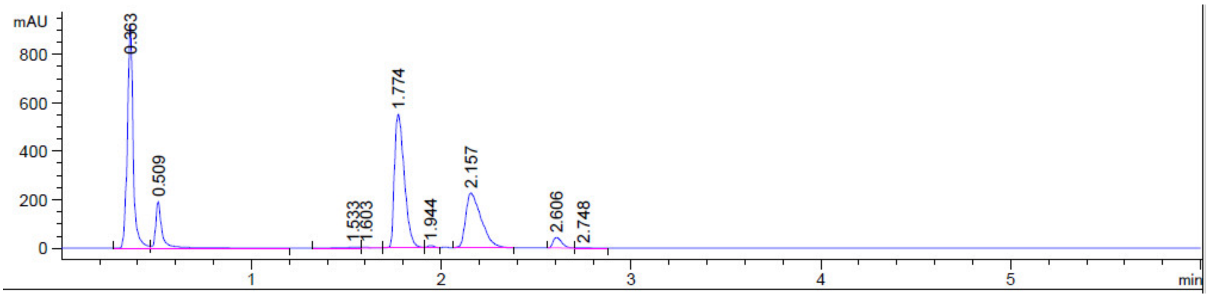

Figure S30. HPLC spectrum for chlorination of G_5W produced by enzymatic halogenation using PyrH. Retention time before and after chlorination are 1.774 min and 2.157 min, respectively. No di and tri substituted product were observed by mass extraction in MS.

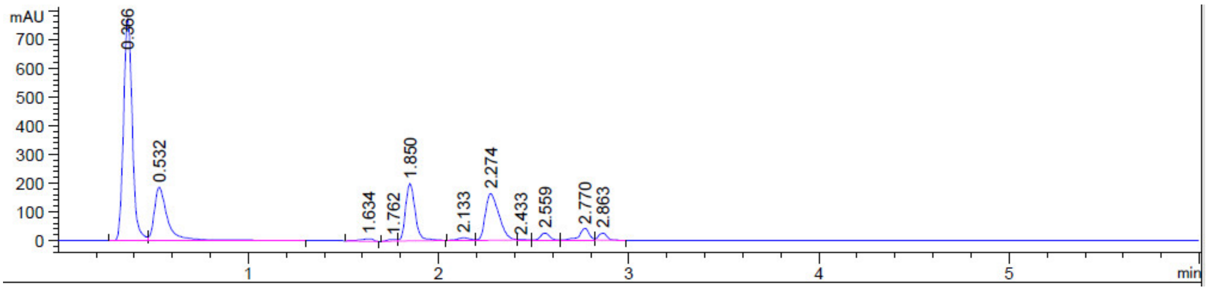

Figure S31. HPLC spectrum for chlorination of SGW produced by enzymatic halogenation using PyrH. Retention time before and after chlorination are 1.850 min and 2.274 min, respectively. No di and tri substituted product were observed by mass extraction in MS.

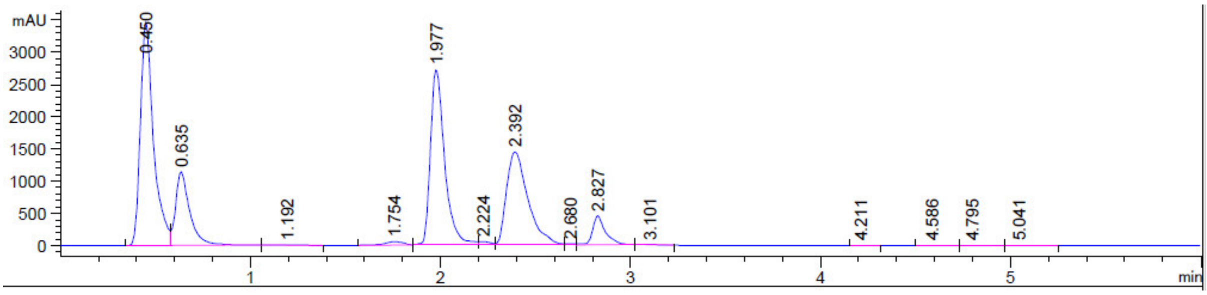

Figure S32. HPLC spectrum for chlorination of G_2SGW produced by enzymatic halogenation using PyrH. Retention time before and after chlorination are 1.977 min and 2.392 min, respectively. No di and tri substituted product were observed by mass extraction in MS.

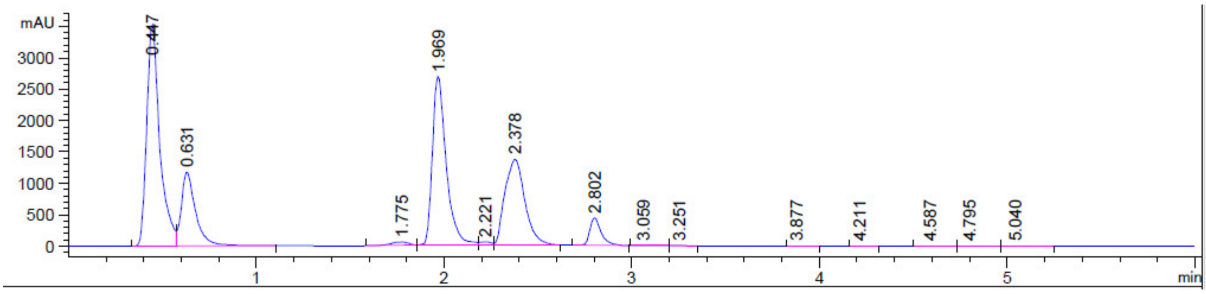

Figure S33. HPLC spectrum for chlorination of G_3SGW produced by enzymatic halogenation using PyrH. Retention time before and after chlorination are 1.969 min and 2.378 min, respectively. No di and tri substituted product were observed by mass extraction in MS.

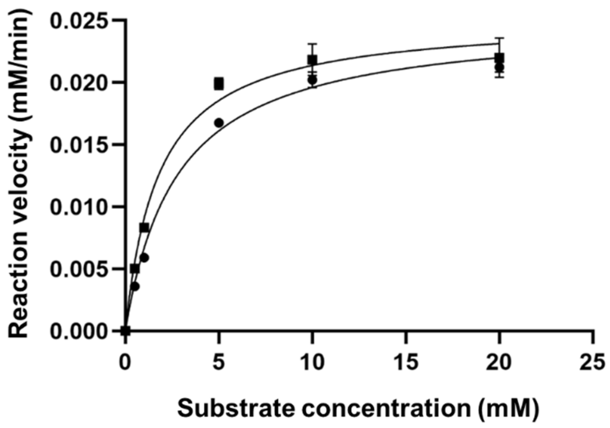

Figure S34. Michaelis-Menten plot for calculating kinetic parameters for chlorination of GGW using WT PyrH (solid circles) and PyrH-Q160N (solid squares). Values represent average ±SD (n=2).

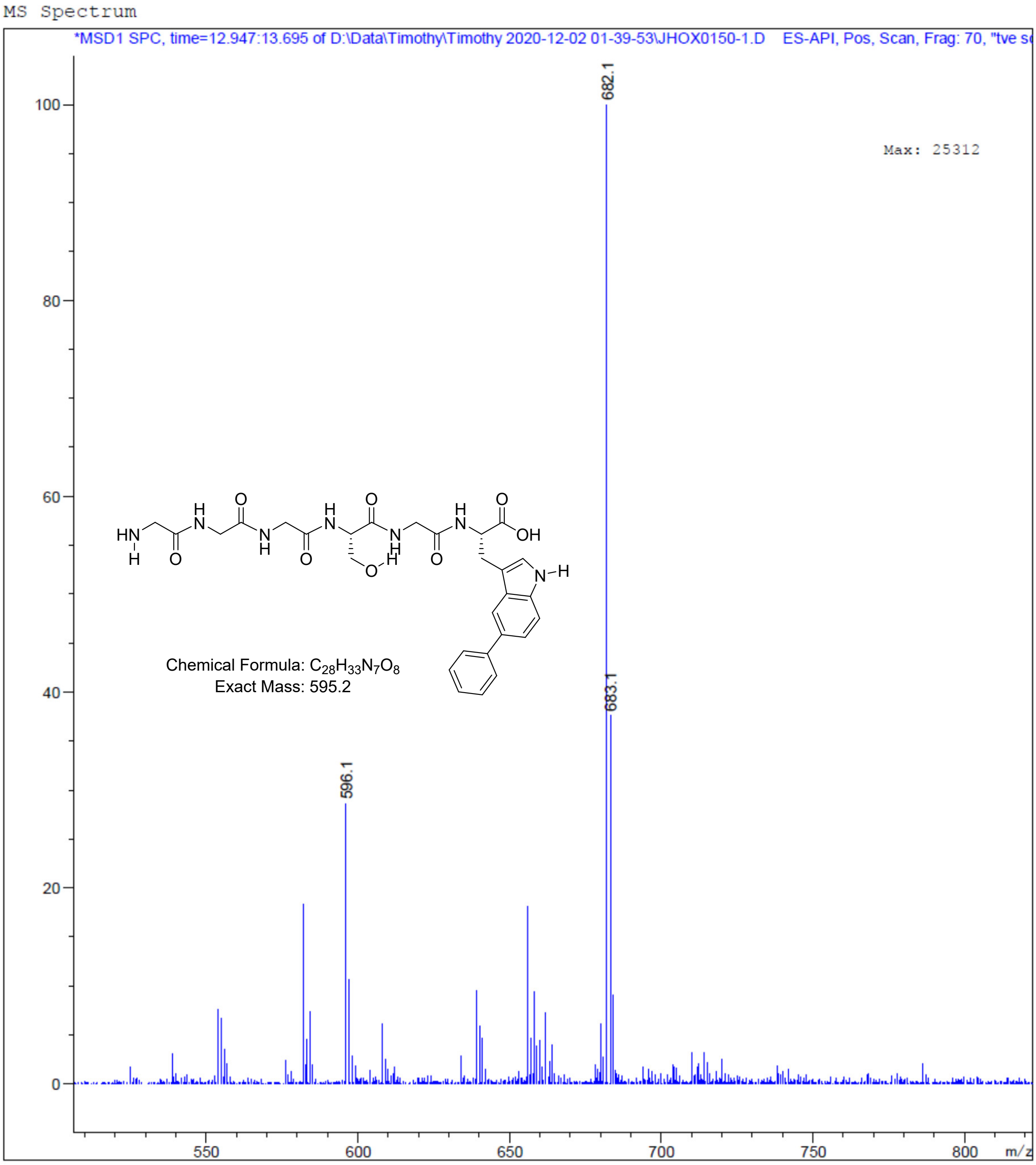

Figure S35. Extracted MS (ESI+) spectrum of phenyl substituted G_3SGW

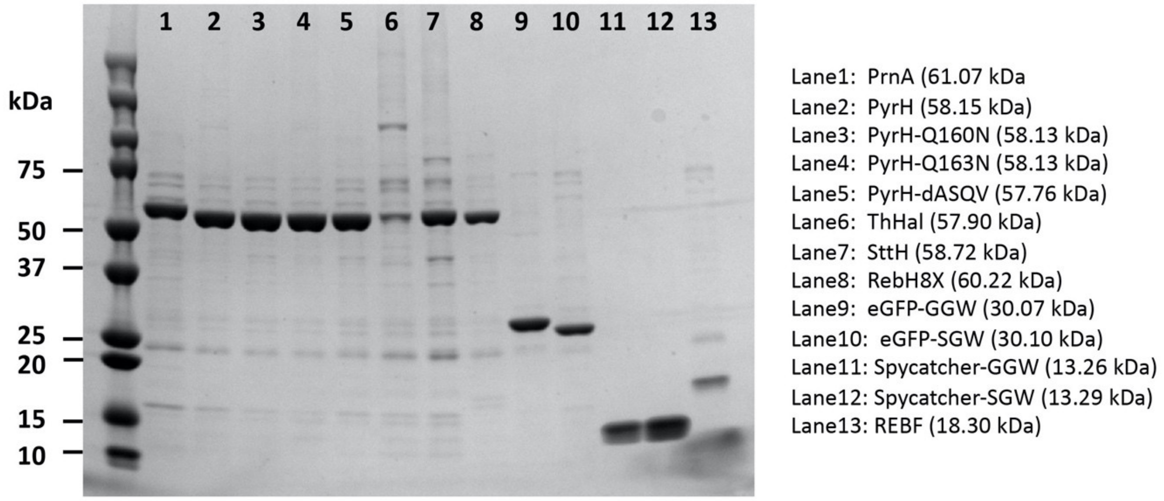

Figure S36. SDS-PAGE analysis of indicated purified proteins.

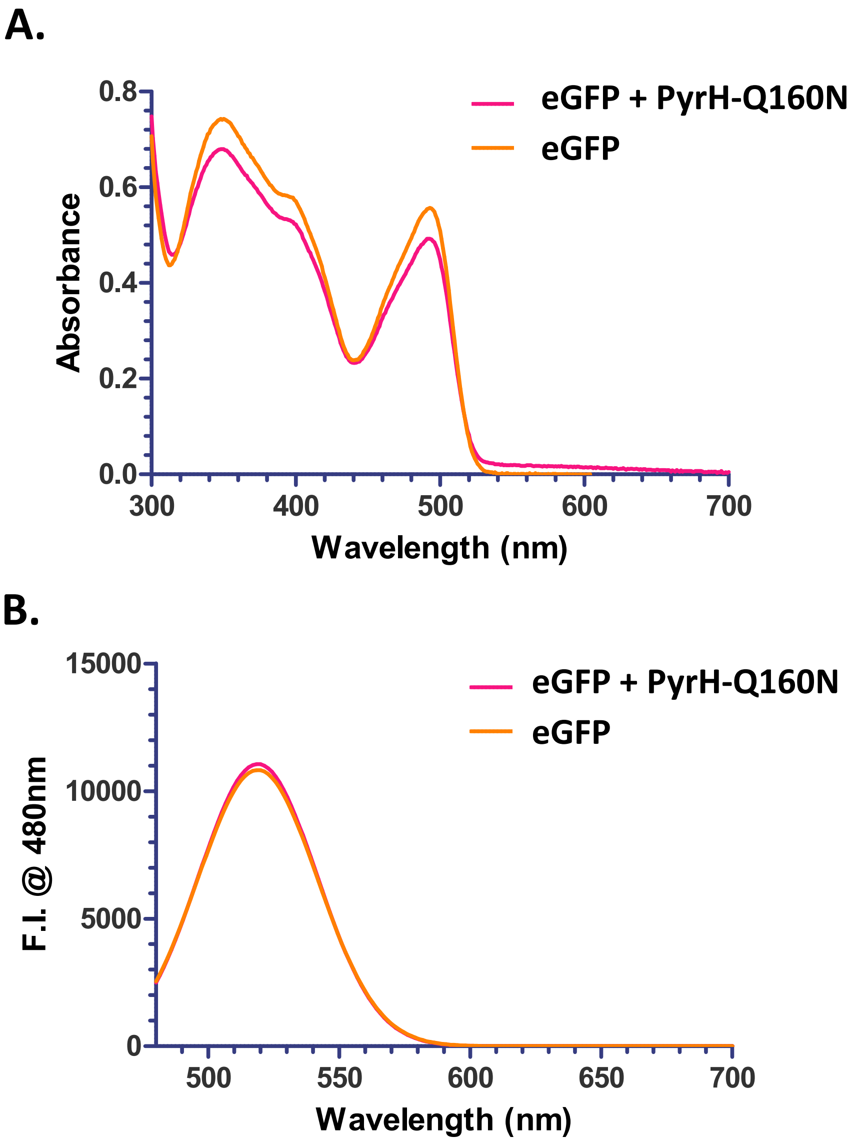

Figure S37. A. Absorbance spectra of eGFP and eGFP halogenated with PyrH-Q160N enzyme (5 mg/mL) in 10 mM phosphate buffer (pH = 7.2). B. Emission spectra of eGFP and eGFP halogenated with PyrH-Q160N enzyme (5 mg/mL) in 10 mM phosphate buffer (pH = 7.2). All spectral measurements (UV–visible absorbance and fluorescence emission) were performed with a PerkinElmer 2104 EnVision Multilabel Plate Reader. UV–visible spectra were recorded between 300 and 700 nm at 1 nm resolution. Ex/Em bandwidths for fluorescence measurements were kept at 8 nm; Ex wavelength was set at 480 nm.
